# Role of Hindmilk in Weight Gain of Preterm Low-Birth-Weight Neonates: A Prospective Comparative Study

**DOI:** 10.7759/cureus.67717

**Published:** 2024-08-25

**Authors:** Jayasrinivas S Gandikota, Shankargouda V Patil, Mallanagouda Patil

**Affiliations:** 1 Pediatrics, Shri B. M. Patil Medical College Hospital and Research Centre, BLDE (Deemed to be University), Vijayapura, IND

**Keywords:** hindmilk, low-birth-weight babies, weight gain, preterm, anthropometry

## Abstract

Background

The significance of low birth weight cannot be overstated when considering the mortality rates during the perinatal and neonatal stages. Babies who were born premature, especially those with extremely low birth weights, have significant health challenges and require adequate feeding to grow and develop well. Specifically, hindmilk is rich in essential nutrients for neonate growth and development. This study aims to evaluate how hindmilk impacts the weight gain and anthropometry (specifically occipitofrontal circumference and length) of preterm low-birth-weight neonates.

Methods

A prospective comparative study was conducted on 148 preterm low-birth-weight neonates admitted to the neonatal intensive care unit of a tertiary care hospital in Vijayapura, part of Northern Karnataka. Informed consent was taken and scrutinized by the Institutional Review Board of BLDE University (approval number: BLDE(DU)/IEC/653/2022-23). The neonates were categorized as Group 1, which received hindmilk, or Group 2, which received composite milk based on the computer-generated block randomization list by the investigator. Weight gain and anthropometry were measured and analyzed using SPSS Statistics version 20 (IBM Corp., Released 2011; IBM SPSS Statistics for Windows, Version 20.0; Armonk, NY: IBM Corp.) at the end of the study.

Results

Group 1 neonates exhibited significantly higher mean values for weight at discharge (1664.22 ± 328.9 grams vs. 1542.33 ± 369.24 grams, p = 0.03), head circumference (31.72 ± 2.52 centimeters (cm) vs. 30.76 ± 4.01 cm, p = 0.04), and length (44.10 ± 2.84 cm vs. 42.23 ± 3.76 cm, p = 0.00) compared to Group 2.

Conclusion

To enhance the growth outcomes of low-birth-weight preterm neonates, selective hindmilk feeding is highly recommended. Hence, it should be adopted in neonatal care to optimize growth and development.

## Introduction

Neonatal care is a serious issue with regard to low birth weight because it is closely related to perinatal and neonatal mortality [1]. For instance, preterm babies with very low birth weights are susceptible to many diseases that emphasize the necessity of early nutrition for their survival and growth [2]. Recent studies have indicated that the nourishment given in the first few weeks of feeding has profound effects on their growth [3].

Human milk, especially from the mother, plays an important role during the neonatal period. It contains essential nutrients as well as biologically active constituents like immunological factors, growth factors, and enzymes, among others, that boost general development and enhance protection against infections [4]. Considering the special physiological requirements of preterm infants, this individualized nutrition is advantageous for them. However, premature babies often experience poor weight gain due to increased energy needs coupled with a limited capacity to take in large quantities.

Milk expressed toward the end of breastfeeding sessions is called hindmilk, and it is higher in fats and calories than foremilk [1]. Similarly, its composition includes necessary long-chain fatty acids, i.e., arachidonic acid and docosahexaenoic acid, responsible for brain development, growth, and vision [2]. The primary aim of undertaking this pilot study was to investigate whether selectively giving premature infants weighing less than 2500 grams only the hindmilk would lead to improved rates of growth.

## Materials and methods

A prospective comparative study was conducted at the neonatal intensive care unit (NICU) of a tertiary care hospital in Vijayapura, part of Northern Karnataka, from June 2022 to December 2023, including preterm low-birth-weight babies. One hundred forty-eight preterm (<37 weeks) newborns with low birth weight (<2500 gm) admitted to the NICU without any complications were included in the study, while those with congenital abnormalities, serious postnatal illnesses (severe birth asphyxia, respiratory distress syndrome, meconium aspiration, congenital anomalies such as tracheoesophageal fistulas and Rh incompatibilities), prolonged respiratory assistance, severe intrauterine growth restriction, maternal human immunodeficiency virus infection, formula feeding, or complications affecting weight gain were excluded. Informed consent was taken and scrutinized by the Institutional Review Board of BLDE University (approval number: BLDE(DU)/IEC/653/2022-23).

Methodology

Newborns were assigned to hindmilk (Group 1) or composite milk (Group 2) feeding groups, with controls being the composite milk group based on the computer-generated block randomization list by the investigator. Composite milk combines the foremilk and hindmilk collected from a mother during a breastfeeding session (composition: fat is 4-5% by volume; this percentage is intermediate between foremilk, which has a lower fat content, and hindmilk, which has a higher fat content; protein is around 1-1.2% by volume; and carbohydrates are approximately 6.5-7% by volume). Gestational age, weight, and anthropometry were recorded for two weeks until discharge. Each baby was nursed in a thermo-neutral environment.

Breast milk collection

Mothers expressed breast milk using a breast pump in clean, labeled containers. Foremilk was identified as the milk gathered during the initial three minutes after the flow started and was stored in labeled containers in the milk bank. A change in color from white to yellow was observed when the hindmilk collection commenced. Hindmilk was defined as the milk collected from this color change until the breast was fully emptied. Containers were sterilized properly. Mothers were counseled on the importance of hindmilk feeding through lactational counselors and trained nursing staff working in the milk bank to ensure adequate compliance with breast milk collection protocol. This support included education on techniques to improve milk production, such as addressing any potential issues like stress or diet that could affect milk supply and effective ways of using breast pumps. Mothers were encouraged to pump frequently, ideally every two to three hours, to stimulate milk production. Mothers were regularly interacted with in the postnatal ward to encourage hindmilk feeding, and awareness was created to avoid myths about breast hygiene.

Feeding protocol

Babies were fed every two hours using nasogastric tubes or spoon-feeding sets. Feed volumes were increased daily by 15 mL/kg up to 200 mL/kg in both groups. This gradual increase helps to ensure that the neonates can tolerate the milk and reduces the risk of gastrointestinal issues. After two weeks, all the newborns were fed full-pumping milk until discharge. Complications like persistent vomiting or apneic attacks led to their exclusion from analysis.

Anthropometry measurement

Babies were weighed daily using a digital neonatal scale for two weeks until discharge. Head circumference and length were measured weekly for two weeks.

Statistical analysis

The data obtained was entered into a Microsoft Excel sheet (Microsoft Corporation, Redmond, WA, USA), and statistical analyses were performed using SPSS Statistics version 20 (IBM Corp., Released 2011; IBM SPSS Statistics for Windows, Version 20.0; Armonk, NY: IBM Corp.). Results were presented as mean, standard deviation counts, percentages, and diagrams. Normally distributed continuous variables between the two groups were compared using an independent sample t-test. For non-normally distributed variables, the Mann-Whitney U test was used. Categorical variables between the two groups were compared using the chi-square test/Fisher's exact test. A p-value of <0.05 was considered statistically significant. All statistics were performed two-tailed.

## Results

The study included 148 preterm and low-birth-weight neonates, evenly distributed between Group 1 (hindmilk) and Group 2 (composite milk). There was no significant difference in maternal age between the groups. However, gestational age was significantly lower in Group 1 (29.43 ± 8.23 weeks) compared to Group 2 (32.03 ± 1.80 weeks) (p = 0.009). Admission head circumference and length were significantly lower in Group 1 (29.52 ± 2.58 cm and 40.53 ± 3.97 cm) compared to Group 2 (30.28 ± 1.73 cm and 42.34 ± 2.77 cm) with p-values of 0.03 and 0.002, respectively. The gender distribution and mode of delivery showed no significant differences between the groups. However, Group 1 had more primiparous mothers (39, 52%) compared to Group 2 (23, 31%) (p = 0.03). APGAR scores at birth were significantly different, with Group 1 having more neonates with higher APGAR scores (8/10) compared to Group 2 (p = 0.04). Thus, there were significant differences in gestational age, admission head circumference, and admission length of the babies between the two study groups (p < 0.05). These differences between the groups were attributed to the randomization process. The baseline characteristics of both groups are summarized in Table 1.

**Table 1:** Comparison of the baseline characteristics between the study groups N: number of neonates p-value <0.05 is considered significant

Baseline characteristics	Group 1 (hindmilk) N=74	Group 2 (composite milk) N=74	p-value
Maternal age (years)	23.97 ± 3.02	24.52 ± 3.69	0.32
Gestational age (weeks)	29.43 ± 8.23	32.03 ± 1.80	0.009
Admission weight (grams)	1621.95 ± 321.87	1611.09 ± 360.61	0.84
Admission head circumference (cm)	29.52 ± 2.58	30.28 ± 1.73	0.03
Admission length (cm)	40.53 ± 3.97	42.34 ± 2.77	0.002
Vaginal delivery	35	38	0.13
Cesarean delivery	39	36	
Gestational age 28-32 weeks	44	37	0.54
Gestational age 32-34 weeks	30	37	
Primigravida	39	23	0.03
Multigravida	35	51	
APGAR at 5 minutes 8/10	26	27	0.38
APGAR at 5 minutes 9/10	45	47	

In terms of anthropometric measurements at discharge, Group 1 had significantly higher mean values for weight (1664.22 ± 328.9 grams vs. 1542.33 ± 369.24 grams, p = 0.03), head circumference (31.72 ± 2.52 cm vs. 30.76 ± 4.01 cm, p = 0.04), and length (44.10 ± 2.84 cm vs. 42.23 ± 3.76 cm, p < 0.01) compared to Group 2.

Table 2 clearly outlines the progression in weight, head circumference, and length from admission through discharge for both groups (length of the stay of neonates in the NICU being two weeks), highlighting significant differences in the growth metrics between the two groups. These findings reinforce the benefits of hindmilk in promoting better growth and development in preterm and low-birth-weight neonates.

**Table 2:** Comparative table of weight and head circumference and length between the study groups at admission and discharge N: number of neonates, mean: mean value of the parameter, SD: standard deviation p-value < 0.05 is considered significant

Parameter	Time point	Group 1 (neonates who received hindmilk) mean ± SD	Group 2 (neonates who received composite milk) mean ± SD	p-value
Weight (grams)	Admission	1621.95 ± 321.87	1611.09 ± 360.61	0.84
	Discharge	1664.22 ± 328.9	1542.33 ± 369.24	0.03
Head circumference (cm)	Admission	29.52 ± 2.58	30.28 ± 1.73	0.03
	Discharge	31.72 ± 2.52	30.76 ± 4.01	0.04
Length (cm)	Admission	40.53 ± 3.97	42.34 ± 2.77	0.002
	Discharge	44.10 ± 2.84	42.23 ± 3.76	0.00

## Discussion

This study investigated the impact of selective hindmilk feeding on growth and health outcomes in preterm and low-birth-weight neonates, highlighting significant differences compared to those receiving composite milk. Our findings demonstrate that neonates receiving hindmilk consistently showed superior growth parameters compared to the composite milk group. Despite a higher prevalence of prematurity in the hindmilk group, these neonates initially presented with comparable admission weights but smaller head circumferences and lengths, potentially reflecting early developmental differences associated with prematurity rather than milk type alone.

In contrast to Ogechi et al.'s [5] findings, which reported similar mean gestational ages and comparable admission weights between groups, our study observed significant differences in gestational age (p = 0.009), as well as smaller head circumferences (p = 0.03) and lengths (p = 0.002) in the hindmilk group. This underscored the influence of demographic differences on baseline characteristics and study outcomes.

Throughout the study period, neonates fed hindmilk consistently exhibited better growth metrics at discharge, with significantly higher weights (p = 0.03), head circumferences (p = 0.04), and lengths (p = 0.00) compared to those receiving composite milk. These findings align with prior studies by Spencer et al. [6], Valentine et al. [7], Slusher et al. [8], and Alshaikh et al. [9], which highlighted rapid weight gain and improved head growth among preterm neonates fed hindmilk, attributed to its higher lipid content.

Additionally, hindmilk-fed neonates experienced fewer complications during the study period, indicating better initial adaptation to extrauterine life and potentially reducing short-term health risks. This supports existing literature indicating hindmilk's benefits for early postnatal development and health outcomes [10,11]. However, neonates with lower APGAR scores at birth exhibited slower weight gain and smaller head circumferences than those with higher APGAR scores. This implies that neonates with lower APGAR scores might need more time before attaining optimal neurodevelopmental outcomes, which could be due to challenges at birth coupled with difficulties adapting to life outside the womb physiologically. Although Shukla et al.’s [12] findings were similar to ours, Costa et al. [13] arrived at a different conclusion. Our research primarily delved into nutritional interventions such as hindmilk feeding; however, these results point out that neonatal health is multifaceted and underscore the need for comprehensive care strategies addressing immediate health issues and developmental needs for all neonates, especially those with compromised conditions at birth. Also, it was observed that babies in the hindmilk group were quieter and slept more peacefully between feedings. This behavior indicates that hindmilk feeding is not only nutritionally adequate but also more satisfying for the neonates, promoting better rest and potentially contributing to their overall growth and development.

Furthermore, analysis by gestational age groups (28-32 weeks and 32-34 weeks) revealed significant differences in weight (p = 0.002), head circumference (p = 0.03), and length (p = 0.03, p = 0.04) at discharge, underscoring the role of milk type in influencing anthropometric outcomes, particularly in very premature neonates.

From admission through discharge, the hindmilk-fed neonates continued to demonstrate better weight gain compared to their counterparts fed composite milk, emphasizing the lasting benefits of hindmilk on ongoing growth and development even after the transition to home care. A study conducted by Alshaikh et al. [9] observed that hindmilk improves head growth in very preterm neonates after discharge, in accordance with the results of our study.

While hindmilk feeding has been extensively studied for its effects on weight gain and head growth, there is limited specific data on its impact on the length of babies. Further studies may be needed to examine the possible effects of hindmilk on neonatal length and to complement existing knowledge on its benefits on weight gain and head circumference [14,15].

To sum up, our study establishes a solid foundation for the advantages of hindmilk to growth and short-term health effects, as well as anthropometric measurements in preterm or low-birth-weight neonates. Enhancing access to hindmilk at medical institutions may enhance infant developmental paths and outcomes on a wide scale. Further studies are needed to examine these longer-term effects and consider other factors that influence neonatal growth and development beyond the neonatal period.

Strengths

The prospective comparative design ensures real-time data collection, thus reducing the recall bias and enhancing reliability. Random assignment to hindmilk and composite milk feeding groups also minimizes selection bias, ensuring valid comparability at baseline. By excluding neonates with conditions affecting weight gain, the study effectively isolates the impact of hindmilk feeding.

Limitations

The limitations of this study include its single-center design, which may limit the generalizability of the results to other clinical settings. In addition, the study focused on short-term outcomes and, therefore, could not assess the long-term effects of hindmilk feeding. Finally, despite efforts to control for confounding variables, factors such as maternal nutrition and socioeconomic status may affect neonatal growth outcomes, but these factors were not fully considered in this study. Inadequate healthcare providers (lactational counselors, trained nursing staff), limited resources (breast pumps, sterilization equipment), busy labor and delivery units, diverse cultural backgrounds, and language barriers are potential challenges for implementing hindmilk feeding in a broader clinical setting.

## Conclusions

This study confirmed that hindmilk had significant benefits for the growth and health of preterm and low-birth-weight neonates, especially those born between 28 and 32 weeks gestation. Despite their higher rate of preterm birth, hindmilk-fed newborns showed better growth parameters at discharge. Prioritizing education for mothers of preterm, low-birth-weight neonates by healthcare professionals to promote hindmilk feeding and addressing the misconceptions and myths to improve the health, well-being, and developmental trajectories of neonates is the need of the hour. Ongoing research and clinical efforts should focus on optimizing strategies to ensure that all at-risk neonates can benefit from this crucial resource.
